# Prediction of Adverse Pregnancy Outcomes Based on Maternal and Pregnancy Characteristics in Triplet Pregnancies

**DOI:** 10.3390/diagnostics15202576

**Published:** 2025-10-13

**Authors:** Gülen Yerlikaya-Schatten, Jasmin Ernst, Florian Heinzl, Sophie Pils, Stephanie Springer

**Affiliations:** Department of Obstetrics and Gynecology, Division of Obstetrics and Feto-Maternal Medicine, Medical University of Vienna, Whäringer Gürtel 18-20, 1090 Vienna, Austria; guelen.yerlikaya-schatten@meduniwien.ac.at (G.Y.-S.); n12026593@students.meduniwien.ac.at (J.E.); florian.heinzl@meduniwien.ac.at (F.H.); sophie.pils@meduniwien.ac.at (S.P.)

**Keywords:** triplet pregnancy, prediction model, adverse pregnancy outcome, uterine artery Doppler, pregnancy-related hypertensive disorders

## Abstract

**Objective**: Multifetal gestations are linked to an increased risk of pregnancy-related hypertensive disorders and other adverse outcomes. The probability positively correlates with the number of fetuses. Therefore, the objective of the study was to assess the use of various maternal and pregnancy-related characteristics for the prediction of adverse pregnancy outcomes in triplet pregnancies, dependent on different possible predictive factors such as maternal age, BMI, assisted reproductive technology (ART), parity, uterine artery Doppler (UtA-PI) measured and chorionicity. **Methods:** This was a screening study in 99 triplet pregnancies to evaluate the risk for adverse pregnancy outcomes for PE, hypertension, fetal growth restriction (FGR), intrauterine fetal death (IUFD), small for gestational age (SGA) and preterm birth below 32 + 0 gestational weeks, dependent on different possible predictive factors. Logistic regression analysis was performed. **Results:** 99 triplet pregnancies were included. Additionally, 58 women (58.6%) developed adverse pregnancy outcomes: FGR 16.2%, SGA (3.0%). Gestational hypertension was observed in 16 pregnancies (16.2%), and preeclampsia was diagnosed in 11 cases (11.1%). Furthermore, 6 pregnancies (6.2%) were complicated by IUFD, and 36 pregnancies (36.4%) resulted in preterm birth before 32 + 0 weeks of gestation. **Conclusions:** Hypertension and PE are common maternal complications in triplet pregnancies. While higher maternal age is a clear predictor of hypertension and PE, a model based on maternal and pregnancy characteristics did not provide sufficient predictive accuracy.

## 1. Introduction

In recent years, the introduction of assisted reproductive technologies has led to an increase in multifetal pregnancies. However, compared to singleton and twin pregnancies, triplet pregnancies are associated with markedly higher risks of both maternal and fetal morbidity. This is reflected, among other indicators, in the significantly higher incidence of gestational hypertension, pre-eclampsia (PE), gestational diabetes mellitus (GDM), and preterm birth among women with triplet pregnancies [1]. Research has consistently shown that multifetal gestations are linked to an increased risk of pregnancy-related hypertensive disorders [2,3,4,5,6]. The probability positively correlates with the number of fetuses carried and affects approximately 6.5% of all singleton pregnancies, 12.7% of twin pregnancies, and 20% of all triplet pregnancies [7]. Other possible complications include intrahepatic cholestasis, anemia, major obstetric hemorrhage, and cesarean section [8,9,10]. As a result, fetal reduction may be considered for patients with higher-order multifetal pregnancies. Fetal reduction has been shown to reduce the incidence of preterm birth (defined as delivery before 32 + 0 weeks of gestation) to approximately 10%; however, it is also associated with an elevated risk of miscarriage [11]. Preventing preterm delivery in triplet pregnancies—and thereby reducing fetal, neonatal, and long-term childhood complications—has been a primary goal of obstetric care. The underlying causes of the increased maternal and fetal complications in triplet pregnancies are not yet fully understood. Recently, an increasing number of studies have examined the potential impact of chorionicity on triplet pregnancies. Intertwin placental vascular anastomoses and shared circulation underlie the development of twin-to-twin transfusion syndrome (TTTS), selective fetal growth restriction (sFGR), acute feto-fetal hemorrhage, and cardiovascular compromise in monochorionic fetuses. It also accounts for the severe neurological damage often seen in surviving twins after the loss of a single fetus. Furthermore, the presence of these vascular anastomoses explains why mono- and dichorionic triplet pregnancies have higher risks of morbidity and mortality compared to trichorionic triplets [12]. The impact of chorionicity on maternal, fetal, and neonatal morbidity and mortality has not yet been fully clarified.

However, unlike in singleton pregnancies, no screening strategies for adverse outcomes, such as preeclampsia screening, have been established for higher-order multifetal pregnancies, limiting the potential of risk stratification. The competing-risks model introduced by Nicolaides assesses the risk of preeclampsia and stratifies pregnancy care by combining maternal factors and biomarkers across the first, second, and third trimesters [13,14,15,16,17]. In the first trimester, maternal blood pressure, uterine artery pulsatility index (UtA-PI) and placental growth factor (PlGF) were used to identify women at high risk of developing preterm PE. At a 10% screen-positive rate, 90% of early-onset PE cases and 75% of preterm PE cases were successfully predicted [18]. Several studies highlight the integration of UtA-PI with maternal factors to enhance predictive accuracy for conditions like preeclampsia and fetal growth restriction [18]. A systematic review showed that UtA-PI, alone or combined with notching, is the most predictive Doppler index [19,20]. Scandiuzzi et al. demonstrated that maternal UtA-Doppler in the first and second trimester is an effective screening method for hypertensive disorders and adverse perinatal outcomes in low-risk pregnancies [20]. Risk stratification in pregnancy is crucial for identifying potential complications. Therefore, UtA-PI Doppler is a valuable tool for predicting adverse pregnancy outcomes in both singleton and multiple pregnancies [21]. Studies indicate that it can effectively identify risks associated with complications, particularly in twin pregnancies, thereby enhancing clinical decision-making [22,23]. Previous studies have assessed the use of uterine artery notching at 20–22 weeks of gestation in twin pregnancies, as well as the combination of notching and UtA-PI for screening preeclampsia. The authors demonstrated that combining uterine artery notching with the highest UtA-PI above the 95th centile provided the best screening performance. The sensitivities for predicting preeclampsia were 50% for notching alone, 45% for the highest UtA-PI alone, and 91% for the combination of notching and the highest UtA-PI [21]. However, there is currently no such data available for triplet pregnancies, despite their increasing incidence. Therefore, the objective of the study was to assess the use of various maternal characteristics and pregnancy-related characteristics as maternal age, body mass index (BMI), parity, UtA-PI and chorionicity for the prediction of adverse pregnancy outcomes in triplet pregnancies.

## 2. Materials and Methods

This was a screening study in 99 triplet pregnancies to evaluate the risk for adverse pregnancy outcomes like PE, hypertension, fetal growth restriction (FGR), intrauterine fetal death (IUFD), small for gestational age (SGA) and preterm birth below 32 + 0 gestational weeks dependent on different possible predictive factors such as maternal age, BMI, assisted reproductive technology (ART), parity, UtA-PI measured at 19 + 0 to 23 + 6 weeks of gestation and chorionicity conducted at the Department of Obstetrics and Gynecology of the Medical University of Vienna, Austria, a tertiary care center with around 2800 deliveries annually. The inclusion criteria comprised women with triplet pregnancies who delivered phenotypically normal fetuses. Exclusion criteria included pregnancies that underwent fetal reduction, were complicated by aneuploidy, or involved major fetal anomalies. Additionally, patients who did not undergo uterine artery Doppler assessment between gestational weeks 19 + 0 and 23 + 6 were excluded from the analysis.

Over a 25-year period, a total of 316 patients with triplet pregnancies were managed at the Medical University of Vienna. Of these, 217 were excluded due to missing data or because they did not meet the inclusion criteria.

The definition of adverse pregnancy outcome included complications such as PE, hypertension, FGR, SGA, IUFD and preterm birth below 32 + 0 gestational weeks.

**Figure 1:**
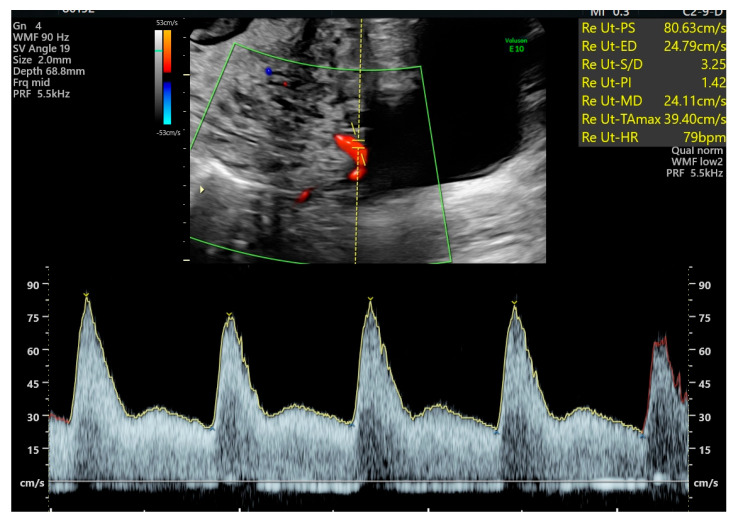
Uterine Artery Doppler: transabdominal measurement with a hint of a notch.

The Due date was calculated based on the first day of the last menstrual period and adjusted to ultrasound findings. Ultrasound examinations were performed using either a Toshiba Xario, a Toshiba Aplio (Canon Medical System Ltd., Ōtawara, Japan), as well as a Voluson E8 Expert system equipped with a transabdominal RAB 4-8-D convex probe or a GE Healthcare Voluson E10 system (GE HealthCare, Zipf, Austria) with a transabdominal RM6C convex probe. Chorionicity was assessed during first-trimester screening, performed between 11 + 0 and 13 + 6 weeks of gestation, in accordance with the criteria established by the Fetal Medicine Foundation (London, UK). All women underwent a fetal anomaly scan between 19 + 0 and 23 + 6 weeks of gestation. During this examination, the presence of notching and the pulsatility index (PI) of both uterine arteries were assessed using transabdominal ultrasound. After identifying the uterine arteries (color flow mapping), a pulsed wave Doppler was conducted at the crossover of the uterine and external iliac arteries. When three similar consecutive waveforms were provided, the presence of notching was determined, and the PI was measured on each side (Figure 1). Ongoing routine monitoring until delivery was performed at intervals of two or four weeks, depending on chorionicity. Routine clinical management after the anomaly scan and until delivery included biweekly visits or every four weeks, depending on the chorionicity. Each visit included an assessment of fetal growth and measurement of the deepest amniotic fluid pocket for each fetus individually. Furthermore, fetal Doppler measurements of the umbilical artery and, when indicated, the middle cerebral artery (MCA) were performed in accordance with ISUOG Guidelines [24]. Demographic characteristics and data on fetal and maternal outcomes were collected from the hospital maternity records (Viewpoint^®^ software version 5, GE Healthcare, Wessling, Germany).

Preeclampsia was defined according to the American College of Obstetricians and Gynecologists (ACOG) as new-onset hypertension (≥140/90 mmHg) presenting after 20 weeks of gestation, confirmed on two separate measurements at least 4 h apart in previously normotensive women, or as severe hypertension (≥160/110 mmHg) accompanied by proteinuria. Proteinuria was characterized as the excretion of ≥300 mg of protein in a 24 h urine collection, a protein-to-creatinine ratio of ≥0.3 mg/dL, or a urine dipstick reading of 2+ (used only when other quantitative methods were unavailable) [25].

In women with new-onset hypertension, even in the absence of proteinuria, the development of any of the following was diagnostic of preeclampsia: platelet count below 100,000/μL, serum creatinine above 1.1 mg/dL, liver transaminases at least twice the upper limit of normal, pulmonary edema, or neurological or visual symptoms. Gestational hypertension was defined as a blood pressure ≥140/90 mmHg occurring after 20 weeks of gestation in the absence of significant proteinuria [26].

Delivery with cesarean section was performed in an uneventful pregnancy around 34/35 weeks of gestation. The study was approved by the Ethical Review Board of the Medical University of Vienna (EK Nr: 2071/2018) and conducted in accordance with the principles of the Declaration of Helsinki. Pseudonymized data from medical records were used, so direct patient participation was not required. Nonetheless, in line with internal hospital standard operating procedures, written informed consent was obtained at patient registration.

### Statistical Analysis

Categorical data are presented as absolute and relative frequencies, while numerical data are summarized using the median and inter-quartile range. In order to investigate the factors associated with adverse pregnancy outcomes, logistic regression analysis was performed. Due to the large number of dependent variables of interest compared to the sample size, we opted to penalize the logistic regression via elastic net. The necessary lambda was chosen as follows: we started with a 5-fold cross-validation and constructed a regularization path.

The optimal lambda, determined based on the pooled mean square error, was first calculated. To adopt a more conservative approach, we then selected the biggest lambda for which the MSE is within one standard error of the optimal lambda for use in our final model. Two models were constructed. We started with the one presented, and then “chunk tested” the uterine artery Doppler variables. Since there was one difference in interpretation between the two, the uterine artery Doppler variables have been dropped in order to obtain a more parsimonious model. Additionally, missing numerical data has been imputed by chained equations (predictive mean matching).

## 3. Results

Out of 99 triplet pregnancies included in this retrospective analysis, a total of 58 women (58.6%) developed adverse pregnancy outcomes. Fetal growth restriction (FGR) was identified in 16 triplet pregnancies (16.2%), and small for gestational age (SGA) in 3 pregnancies (3.0%), each involving at least one fetus per pregnancy. Gestational hypertension was observed in 16 pregnancies (16.2%), and preeclampsia was diagnosed in 11 cases (11.1%). Furthermore, 6 pregnancies (6.2%) were complicated by IUFD, and 36 pregnancies (36.4%) resulted in preterm birth before 32 + 0 weeks of gestation. Clinical characteristics of all included pregnancies and the subgroups are presented in Table 1.

As seen in Table 2, the median mean UtA-PI of the left and right uterine artery in gestational weeks 19 + 0 − 23 + 6 was 0.74 (0.64–0.89) in women without adverse pregnancy outcomes and 0.76 (0.64–0.93) in women with adverse pregnancy outcomes (*p* = 0.8064). Even in the evaluation of the mean UtA-RI, the minimum and maximum UtA-PI, and the presence of notching, no significant differences were observed.

Chorionicity distribution was as follows: 54 (54.5%) trichorionic, 42 (42.4%) dichorionic, and 3 (3.0%) monochorionic pregnancies. Patient characteristics and pregnancy outcomes according to chorionicity are presented in Table 3. Due to the small number of cases, monochorionic triplet pregnancies were excluded from the subgroup analysis. The mean age was 32 years (27.3–36) in the trichorionic triplet group, compared to 34 years (29.5–37) in the dichorionic group. In our cohort, maternal age was predominantly < 40 years. ART is typically performed for women aged 20 to 40, as success rates decline significantly with age. Women above 40 are less frequently represented in triplet pregnancies, likely due to lower conception rates, comorbidities, and stricter ART protocols. Only a few women above 40 years were identified in our cohort, which may reflect a selection effect: that we may have excluded a proportion of patients due to incomplete data. Nevertheless, it is assumed that women over 40 are less likely to conceive, and due to comorbidities or stricter ART protocols, they are less frequently represented in triplet pregnancies. A significantly lower BMI (*p* = 0.0120) was observed in patients with dichorionic triplets (22.6, 20.6–24.8) compared to trichorionic triplets (24.98, 22.3–29.1). Assisted reproductive techniques were more frequently used in trichorionic triplet pregnancies, with only in vitro fertilization reaching statistical significance. With the exception of TTTS, none of the complications showed a higher incidence in either group. In total, 7 patients developed TTTS, of whom 3 had to undergo laser treatment. TTTS is a typical complication of monochorionic pregnancies, and the fact that there were only 3 TTTS with laser treatment in the entire cohort, we do not assume that it has an influence on the assessment of outcomes, and therefore we chose not to exclude those cases. PE occurred in 6 patients (11.1%) with trichorionic triplet pregnancies and in 4 patients (9.5%) with dichorionic triplet pregnancies (*p* = 0.8489). Likewise, gestational age at delivery was comparable between the groups. The median gestational age at delivery was 227 days (216.25–238.0) in the trichorionic group, and 230.5 days (208.5–237.75) in the dichorionic cohort (*p* = 0.8942).

Uterine artery Doppler indices according to chorionicity are presented in Table 4. The median mean UtA-PI was 0.73 (0.65–0.88) in women without dichorionic pregnancies and 0.81 (0.65–0.94) in women with trichorionic pregnancies (*p* = 0.2842). No significant differences in uterine artery Doppler indices were observed between the two groups. A trend toward statistical significance was observed only for the minimum UtA-PI between the two groups (*p* = 0.0511).

While birth weight was lower in dichorionic triplet pregnancies, the difference was not statistically significant (*p* = 0.0906).

Logistic regression analysis revealed that adverse pregnancy outcome was not well predicted by a combination of factors, including maternal age, BMI, parity, ART and chorionicity with an area under the ROC curve of 68.3% (Table 5 and Table 6). Among these, increased maternal age emerged as the most significant risk factor. The median age was significantly different between individuals with and without adverse pregnancy outcomes (*p* = 0.0455). In contrast, no significant difference was observed in BMI between the groups (*p* = 0.1734).

Overall, the results show that hypertension and preeclampsia are common maternal complications in triplet pregnancies. While higher maternal age is a clear predictor of adverse pregnancy outcome, the commonly available risk factors do not adequately assess the risk in this population.

A model combining age, BMI, IVF, chorionicity, and parity achieved a higher AUC (0.6829) and lower log loss and Brier scores than the null model, indicating improved predictive performance for adverse pregnancy outcomes. However, the *p*-value (0.0836) indicates that the model’s predictive ability is not statistically significant.

## 4. Discussion

In our study, we investigated if there are different maternal and pregnancy characteristics for the prediction of adverse pregnancy outcome in triplet pregnancies. While higher maternal age was identified as a predictor for adverse pregnancy outcomes in triplet pregnancies, BMI, ART, parity, UtA-PI and chorionicity did not demonstrate sufficient predictive value. To our knowledge, this is the first study with an attempt to provide a prediction model for triplet pregnancies for risk stratification.

In numerous studies, it was proven that the best and most useful approach for estimation of individual patient-specific risk of delivery with PE is by a combination of maternal factors and biomarkers, obtained either individually or in combination at any stage in pregnancy, using the competing-risks model developed by Nicolaides et al. [27,28]. In their screening study the risk for PE increased with maternal age, BMI, Black and South Asian racial origin, previous pregnancy with PE, conception by in vitro fertilization (IVF) and a medical history of chronic hypertension, type 2 diabetes mellitus as well as systemic lupus erythematosus (SLE) or antiphospholipid syndrome (APS). Furthermore, an inverse correlation was observed between the multiple of the median (MOM) values of the UtA-PI and maternal blood pressure with gestational age at delivery. Which leads to the conclusion that screening by using maternal characteristics, UtA-PI and maternal blood pressure can identify 90% of patients with early-onset PE (diagnosis before 34 weeks) [28]. Our cohort showed only partial alignment with these factors, with maternal age being the most prominent distinguishing variable. Although UtA-PI Doppler has been described as a valuable tool for predicting adverse pregnancy outcomes in both singleton and multiple pregnancies, its utility is particularly notable in identifying risks associated with complications in twin pregnancies [21,23]. Our findings suggest that adverse pregnancy outcomes, such as hypertension, could be moderately predicted by using a combination of maternal factors, including maternal age and BMI, parity, ART and chorionicity. However, the predictive value did not differ significantly when comparing the lowest, mean, and highest PI measurements of the uterine artery Doppler. In a previous study involving a cohort of 423 twin pregnancies, a high incidence of PE was related to elevated values of the highest UtA-PI [29]. A subsequent study with 380 twin pregnancies assessed the use of uterine artery notching, as well as the highest, lowest, and mean PI, both individually and in combination with notching, for screening preeclampsia. The authors concluded that the highest sensitivity and specificity for predicting PE were achieved by using a combination of uterine artery notching and the highest PI [21]. When the highest PI was combined with notching, sensitivity rates of nearly 90% could be achieved. Among all evaluated parameters, notching showed the highest sensitivity for the prediction of preeclampsia, early-onset preeclampsia, and fetal growth restriction (FGR). Therefore, incorporating uterine artery notching into the evaluation significantly enhanced the predictive accuracy. However, our data could not show this connection.

Chorionicity has been suggested to be a key determinant of perinatal and maternal outcomes [29]. In our study population, chorionicity did not significantly influence the diagnosis regarding preeclampsia and adverse pregnancy outcome. The literature provides inconsistent conclusions regarding chorionicity and pregnancy outcome in triplet pregnancies. However, a recent meta-analysis revealed that intrauterine mortality is lower in trichorionic-triamniotic (TCTA) pregnancies compared to non-TCTA pregnancies [28,29,30]. Monochorionicity is assumed to increase the risk of morbidity and mortality in triplet pregnancies [31], compared to TCTA pregnancies [31]. However, the impact of chorionicity on the prevalence of maternal, fetal, and neonatal morbidity and mortality has not yet been fully elucidated. Our findings may be partially explained by the underlying pathophysiological mechanisms of a shared placenta, which is known to increase the risk of complications including twin-to-twin transfusion syndrome (TTTS), intrauterine growth restriction (IUGR), anemia–polycythemia sequence, and cardiovascular compromise in monochorionic fetuses [31,32,33]. The same conclusion is drawn by Claverol et al. Triplet pregnancies with a monochorionic pair showed a higher risk of obstetric, fetal and neonatal morbidity and mortality [34]. Newborns with a monochorionic component were more frequently born prematurely before 34 weeks of gestation, had lower birth weights and were more likely to have an APGAR score below 7 at 5 min, as well as an increased incidence of respiratory distress syndrome and overall higher composite neonatal morbidity. The authors conclude that a shared circulation subsequently led to a higher number of complications and therefore earlier delivery [34]. The greater degree of prematurity is likewise linked to lower birth weight and to the primary neonatal complications identified. Another study of 125 triplet pregnancies reported similar findings [12]. DCTA triplets were associated with higher rates of early and extreme preterm delivery. A history of preterm birth was also identified as a risk factor for early preterm delivery in triplets. Additionally, the neonatal death rate was higher in this group [12]. Triplet pregnancies carry a high risk of pregnancy-related complications, particularly influenced by chorionicity, as previously mentioned [31,33]. In addition to chronicity, maternal age is an important factor that significantly influences both maternal and fetal outcomes. A study by Blickstein et al. developed a simple scoring system based on pre-pregnancy maternal characteristics to identify women at higher risk of adverse outcomes in triplet pregnancies.

The maternal characteristics included in the scoring system were parity (nulliparous or multiparous), maternal height (in centimeters), and maternal age. In total, 2887 triplet sets were analyzed according to the following risk factors (nulliparity, height <165 cm, and age <35 years) [35]. Surprisingly, the study showed that triplets born to older mothers > 35 years had better outcomes than triplets born to younger mothers. Nevertheless, the result must be interpreted in the appropriate context. It is important to note that women who conceive through in vitro fertilization (IVF) tend to be older, more deliberate in their decision to conceive, and often receive higher standards of prenatal care.

Furthermore, these women are often financially independent and may have better healthcare coverage. In contrast, subgroups less likely to conceive using IVF tend to have a higher risk of very preterm birth, very low birth weight, and increased perinatal mortality as maternal age rises [35]. Contrary to this, Fenessy et al. showed that triplets conceived via IVF had similar outcomes to those conceived spontaneously in terms of gestational age at delivery, fetal malformation, fetal and neonatal death, with no significant difference in maternal age between the groups (32.9 years for the IVF group vs. 31.6 years for the spontaneous group) [36]. Although mortality risks associated with premature rupture of membranes (PROM) were higher across all maternal age categories (>40, 30–39, 20–29 and <20 years), these differences did not reach statistical significance [37]. Another recent study showed that the risk of poor outcomes is more closely related to the number of fetuses than to maternal age, although a significant increase in risk was observed from age 40 onwards [38]. Smith et al. compared the impact of fetal number and maternal age on the risk of hypertensive disorders of pregnancy (HDP). Of 70,417 women, 12% developed HDP. Multifetal pregnancies (twin and triplet gestations) increase the risk of HDP compared to singletons, regardless of maternal age. The risk of HDP did not significantly increase with maternal age when the number of fetuses was similar [38].

In contrast to the findings in the literature regarding chorionicity and maternal age, our results suggest a different conclusion. In our study population, maternal age was the primary predictor of HDP, while commonly used risk factors did not adequately assess the risk of preeclampsia in this group.

Our results were unexpected and surprising. One possible explanation is that the results could be false negatives, since the characteristics examined—both maternal and biochemical markers—have been shown to clearly influence outcomes in singleton and twin pregnancies, as described above. Therefore, a limitation of the study could be its retrospective design, which may have introduced bias due to incomplete data.

Nevertheless, the main strength of this study is the relatively high number of triplet pregnancies, although probably for a functioning prediction model with all the given factors, one would need significantly more triplet pregnancies. However, we are the first to attempt to create a prediction model and show that there are factors that influence adverse pregnancy outcomes. Although preterm birth is the predominant adverse outcome in triplet pregnancies, its high incidence is already well established. In contrast, the occurrence and predictors of HDP and PE in triplet pregnancies remain less well defined, and no validated risk models are available. Our focus on HDP and PE, therefore, addresses an important need, highlighting complications that are not only clinically relevant but also potentially amenable to preventive strategies and tailored monitoring.

Algorithms should be derived from multivariable logistic regression analyses that combine maternal characteristics with biophysical and biochemical markers to develop individualized risk assessments for each adverse pregnancy outcome. Furthermore, Guideline bodies increasingly recommend consideration of low-dose aspirin for multifetal gestations at elevated risk of preeclampsia [39]; however, evidence in higher-order multiples (triplets) is limited. Therefore, the potential modifying effect of prophylactic medications on uterine Doppler indices and hypertensive outcomes warrants attention in future studies and when applying risk models to clinical practice.

## 5. Conclusions

The study concludes that HDPs are common maternal complications in triplet pregnancies. While higher maternal age is a clear predictor, a model based on maternal and pregnancy characteristics did not provide sufficient predictive accuracy for HDP and other adverse pregnancy outcomes, despite the significantly increased risk of hypertension and PE in women with triplet pregnancies. It is important to consider that triplet pregnancies are inherently delivered earlier, which may affect the model’s performance.

## Figures and Tables

**Table 1:** Patient Characteristics.

Variable	All (*n* = 99)	Control Group (*n* = 41)	Adverse Pregnancy Outcome Group (*n* = 58)	*p*-Value
Age, y, *	33 (28–36)	31 (28–34)	34 (28–37)	0.0455
BMI, kg/m^2^, *	23.5 (20.9–26.3)	24.0 (21.4–28.9)	22.9 (20.9–25.9)	0.1734
Gravidity, *	2 (1–2)	2 (1–2)	1.5 (1–2)	0.8151
Parity, *	0 (0–1)	0 (0–1)	0 (0–1)	0.3797
Smoking, †	12 (12.6)	7 (17.5)	5 (9.1)	0.2798
ART, †	74 (74.8)	30 (73.2)	44 (75.9)	0.7714
Chorionicity, †				0.2614
MC	3 (3.0)	0 (0)	3 (5.2)	
DC	42 (42.4)	16 (39)	26 (44.8)	
TC	54 (54.5)	25 (61)	29 (50)	
Amnioticity, †				0.8274
DA	3 (3)	1 (2.4)	2 (3.5)	
TA	96 (97)	40 (97.6)	56 (96.6)	
FGR, †	16 (16.2)	0 (0)	16 (27.6)	0.0002
SGA, †	3 (3.0)	0 (0)	3 (5.2)	0.1587
IUFD, †	6 (6.1)	0 (0)	6 (10.3)	0.0389
GDM, †	17 (19.1)	97 (17.1)	10 (20.8)	0.8906
TTTS, †	7 (7.1)	0 (0)	7 (12.1)	0.0228
Gestational Hypertension, †	16 (16.2)	0 (0)	16 (27.6)	0.0002
Preeclampsia, †	11 (11.1)	0 (0)	11 (19)	0.0033
Preterm < GA 32 + 0, †	36 (36.4)	0 (0)	36 (62.1)	0.0000
GA at delivery, d, *	227 (211.5–238)	237 (229–240)	216 (197–231)	0.0000

* median (interquartile range), † number (percent). y = year, BMI = body mass index; kg/m^2^ = kilogram per square meter, ART = assisted reproductive technology, MC = monochorionic, DC = dichorionic, TC = trichorionic, DA = diamniotic, TA = triamniotic, FGR = fetal growth restriction, SGA = small for gestational age, IUFD = intrauterine fetal death, GDM = gestational diabetes mellitus, TTTS = twin-to-twin-transfusion syndrome, GA = gestational age, d = days. (Monochorionic pregnancies (MC) (*n* = 3) are shown for completeness but were excluded from any analyses and predictive models due to small sample size).

**Table 2:** Uterine artery Doppler measurements.

Variable	All (*n* = 99)	Control Group (*n* = 41)	Study Group (*n* = 58)	*p*-Value
Mean UtA-PI, *	0.75 (0.64–0.91)	0.74 (0.64–0.89)	0.76 (0.64–0.93)	0.8064
Mean UtA-RI, *	0.5 (0.45–0.55)	0.48 (0.44–0.55)	0.5 (0.42–0.56)	0.8506
Minimum UtA-PI, *	0.65 (0.54–0.81)	0.65 (0.59–0.81)	0.66 (0.54–0.8)	0.7384
Maximum UtA-PI, *	0.89 (0.7–1.01)	0.89 (0.7–1.01)	0.88 (0.7–1.01)	0.7981
Notching left UtA, †	6 (6.2)	3 (7.5)	3 (5.3)	0.8506
Notching right UtA, †	2 (2)	0 (0)	2 (3.5)	0.3396

* median (interquartile range) of the left and right uterine artery, † number (percent). UtA = uterine artery, PI = pulsatility index, RI = resistance index, (Monochorionic pregnancies (MC) (*n* = 3) are shown for completeness but were excluded from all analyses and predictive models due to small sample size.).

**Table 3:** Characteristics and outcomes depending on chorionicity.

Variable	Dichorionic (*n* = 42)	Trichorionic (*n* = 54)	*p*-Value
Age, y, *	34 (29.5–37)	32 (27.3–36)	0.2530
BMI, kg/m^2^, *	22.6 (20.6–24.8)	24.98 (22.329.1)	0.0120
Gravidity, *	1 (1–2)	2 (1–2.8)	0.3920
Parity, *	0 (0–0.8)	0 (0–1)	0.1526
Smoking, †	5 (12.5)	6 (11.5)	0.9619
IVF, †	33 (78.6)	31 (57.4)	0.0316
ICSI, †	7 (16.7)	4 (7.4)	0.1641
Stimulation, †	14 (33.3)	14 (25.9)	0.5642
TTTS, †	7 (16.7)	0 (0)	0.0018
FGR, †	9 (21.4)	7 (13)	0.3436
SGA, †	3 (7.1)	0 (0)	0.0525
IUFD, †	4 (9.5)	2 (3.7)	0.2768
GDM, †	7 (19.4)	10 (20)	1.0000
Gestational Hypertension, †	6 (14.3)	9 (16.7)	0.7970
Preeclampsia, †	4 (9.5)	6 (11.1)	0.8489
GA at delivery, d, *	230.5 (208.5–237.75)	227 (216.25–238)	0.8942

* median (interquartile range), † number (percent). y = year, BMI = body mass index; kg/m^2^ = kilogram per square meter, IVF = in vitro fertilization, ICSI = Intracytoplasmic Sperm Injection, TTTS = twin-to-twin-transfusion syndrome, FGR = fetal growth restriction, SGA = small for gestational age, IUFD = intrauterine fetal death, GDM = gestational diabetes mellitus, GA = gestational age, d = days; monochorionic triplets were excluded from this analysis due to the small number.

**Table 4:** Uterine artery Doppler measurements depending on chorionicity.

Variable	Dichorionic (*n* = 42)	Trichorionic (*n* = 54)	*p*-Value
Mean UtA-PI, *	0.73 (0.65–0.88)	0.81 (0.65— 0.94)	0.2842
Mean UtA-RI, *	0.49 (0.45–0.53)	0.52 (0.46–0.55)	0.2958
Minimum UtA-PI, *	0.64 (0.53–0.74)	0.7 (0.59–0.83)	0.0511
Maximum UtA-PI, *	0.86 (0.73–1.01)	0.9 (0.7–1.02)	0.8160
Notching left UtA, †	2 (4.9)	4 (7.4)	0.6307
Notching right UtA, †	2 (4.9)	0 (0)	0.1102

* median (interquartile range), † number (percent). UtA = uterine artery, PI = pulsatility index, RI = resistance index; monochorionic triplets were excluded from this analysis due to the small number.

**Table 5:** Elastic net logistic regression for adverse pregnancy outcome.

Variables	Coefficients
(Intercept)	−0.0013
Age	0.0169
BMI	−0.0080
ART	0.0000
Chorionicity	0.0000
Parity	0.0000
Smoking	0.0000

BMI = body mass index; ART = assisted reproductive technology.

**Table 6:** The model benchmarks for the predication of adverse pregnancy outcomes.

Model	AUC	Log Loss	Brier	BIC	AIC	Deviance	*p*-Value
Null model	0.5000	0.6755	0.2412	134.2535	131.6892	132.8128	
Age + BMI + ART + Chorionicity + Parity	0.6829	0.6174	0.2168	150.4821	132.5317	118.5317	0.0836

BMI = body mass index, ART = assisted reproductive technology.

## Data Availability

The data presented in this study are not publicly due to privacy restrictions, but available on request from the corresponding author.

## Abbreviations

The following abbreviations are used in this manuscript:

PE	Pre-eclampsia
GDM	Gestational Diabetes Mellitus
TTTS	Twin-to-twin transfusion syndrome
FGR	Fetal Growth Restriction
sFGR	Selective Fetal Growth Restriction
UtA-PI	uterine atery pulsatility index
PlGF	placental growth factor
BMI	Body mass index
IUFD	Intrauterine fetal death
SGA	Small for gestational age
ART	Assisted reproductive technology
IUGR	Intrauterine growth restriction
IVF	In vitro fertilization
PROM	premature rupture of membranes
HDP	hypertensive disorders of pregnancy
